# Prevalence and genetic diversity of *Anaplasma phagocytophilum* in wild small mammals from western Yunnan province, China

**DOI:** 10.3389/fvets.2024.1472595

**Published:** 2024-10-30

**Authors:** Jun-jie Zhu, Hong-ze Zhang, Ru-dan Hong, Dan Yu, Mei Hong, Zheng-xiang Liu, Dong-mei Li, Jia-xiang Yin

**Affiliations:** ^1^School of Public Health, Dali University, Dali, China; ^2^Yunnan Institute of Endemic Disease Control and Prevention, Dali, China; ^3^State Key Laboratory for Infectious Disease Prevention and Control, Collaborative Innovation Center for Diagnosis and Treatment of Infectious Diseases, National Institute for Communicable Disease Control and Prevention, Chinese Center for Disease Control and Prevention, Beijing, China

**Keywords:** *Anaplasma phagocytophilum*, small mammals, genetic diversity, Yunnan province, infection rate

## Abstract

*Anaplasma phagocytophilum* (*A. phagocytophilum*) is an emerging zoonotic pathogen causing human granulocytic anaplasmosis, linked to small mammal reservoirs that harbor various zoonotic pathogens, underscoring their importance in public health and ecology. This study seeks to determine the prevalence of *A. phagocytophilum* in small mammals using PCR, then sequence and genotype positive samples, and assess infection risk factors. Small mammals were seasonally captured and a nested polymerase chain reaction (nested-PCR) was conducted targeting the 16S rRNA gene on spleen samples to detect *A. phagocytophilum* infection from three counties in western Yunnan province, China. Positive samples were sequenced and genotyped, revealing genetic diversity and regional clustering of the pathogen. A total of 1,605 small mammals belonging to 30 species, 18 genera, 6 families, 3 orders were captured seasonally and screened in this region, yielding a 0.93% infection rate with *A. phagocytophilum* (15/1605). Significant variations in infection rates were observed across different species, counties, and habitats. The 16Sr RNA genes of *A. phagocytophilum* were categorized into two distinct clades, indicating notable genetic diversity. The identification of genetic variants in spleen samples underscores the potential public health risk and the critical importance of the One Health approach in disease surveillance. Our findings emphasize the necessity for continuous monitoring and highlight the value of nested-PCR testing on spleen samples for accurate prevalence assessment.

## 1 Introduction

*Anaplasma phagocytophilum* (*A. phagocytophilum*), belonging to the order Rickettsiales, family Anaplasmataceae, genera *Anaplasma*, is a zoonotic tick-borne pathogen transmitted by infected ticks (1, 2). *A. phagocytophilum* primarily infects human granulocytes, such as neutrophils, leading to human granulocytic anaplasmosis (HGA) (3). As a zoonotic disease, the rapid increase of Anaplasmosis prevalence in Europe, America, Africa, and Asia, has aroused widespread concern (4–8). Since clinical signs are unspecific and animals infected with *A. phagocytophilum* may not show any clinical signs at all, diagnosis of granulocytic anaplasmosis can be challenging. In China, seroprevalence studies have indicated that infections with *A. phagocytophilum* in humans have been documented across several provinces. And a study showed that 20% of the tested individuals (64/323) were positive to *A. phagocytophilum* and that the incidence was higher in male (22%) than female (16%) (9). However, it is plausible that the actual number of reported cases is underestimated (9–11).

Persistent infection in reservoir hosts is crucial for sustaining the natural transmission cycle of *A. phagocytophilum* and defining its infection range (10). This pathogen has been identified in a wide range of animal hosts, including dogs, cats, and small mammals (12–15). Small mammals, in particular, serve as reservoirs for numerous zoonotic pathogens, underscoring their importance in public health and ecological research. In general, small mammals, especially in the USA, are important reservoirs of the *A. phagocytophilum* strain pathogenic to humans. And their short lifespan is likely to reduce their epidemiological importance as reservoir hosts, which remains hotly debated (11). Therefore, it is crucial to monitor the infection of *A. phagocytophilum* in small mammals as a potential threat to the health of humans, domestic and wild animals.

Yunnan province is characterized by a complex terrain with subtropical plateau monsoon, maintains a high biodiversity of small mammals that may play important role on *A. phagocytophilum* transmission. However, few surveillance studies have been conducted in this region. The available data are limited to investigations on *Anaplasma* infection in domestic in the northwestern of Yunnan (16). This study aims to detect the *A. phagocytophilum* infection in wild small mammals, characterize their genotypes, and evaluate the environmental factors of *A. phagocytophilum* infection on small mammal from three counties in western Yunnan province.

## 2 Materials and methods

### 2.1 Sampling sites and small mammals trapping

**Figure 1:**
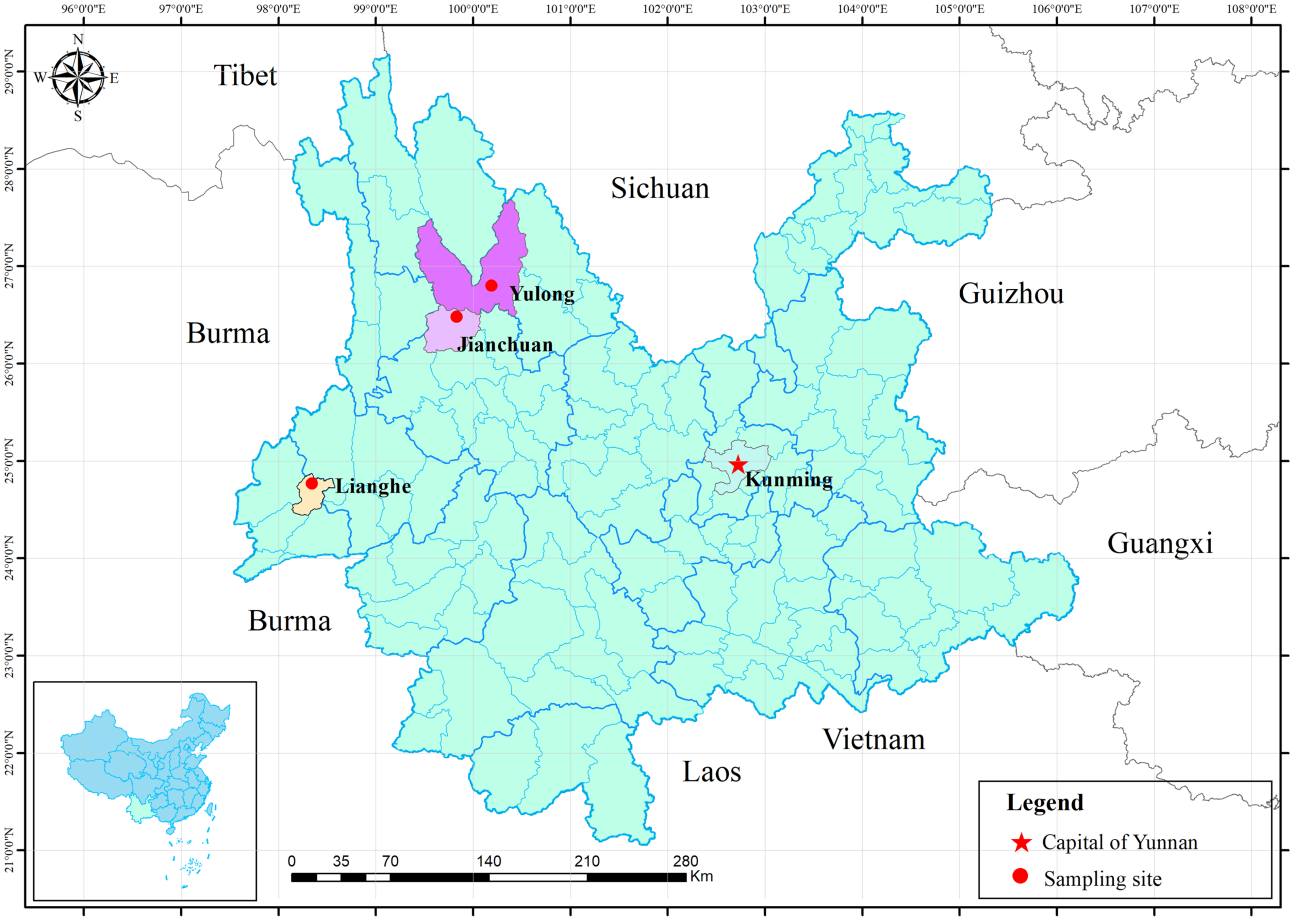
Location of three sampling sites in Yunnan province. Three red dots represent three sampling counties. The red five-pointed star represents Kunming, the capital of Yunnan province.

Using the convenient sampling method, seven villages or towns were selected as sampling sites: three from Lianghe (Mangdong, Hexi and Nangsong), two from Jianchuan (Shilong and Dongshan) and one from Yulong (Wenbi Mountain). The location of three sampling sites was drawn with ArcGIS 10.5 (Figure 1). In each season, small mammals were captured seasonally by snap traps (15 × 8 cm, Metal rat trap clip, China) and peanut as bait set at woodland, cultivated land, thickets in 57 sample locations from seven villages or towns, with a total of 200 snap traps per sample location were placed for three consecutive nights during December 2015 and October 2016. Mammal species were identified according to morphological characteristics in the local Centers for Disease Control (CDC) laboratory and a necropsy was performed, with recordings of gender, habitats and sampling seasons. Spleen tissue samples from each specimen were collected using aseptic techniques and stored at −40°C for DNA extraction.

### 2.2 DNA extraction and nested polymerase chain reaction (nested-PCR) analysis

DNA was extracted from spleen issue using the magnetic beads tissue/cell genome DNA isolation kit (AU19014, Beijing BioTeke Corporation, China) according to manufacturer’s instructions. DNA concentrations were determined by the ultra-micro nucleic acid ultraviolet tester (NanoDrop 1,000, Thermo Fisher Scientific, USA). As the extract concentration was ≥50 μg/mL, and A260/A280 was 1.8 to 2.0, this template was selected for detection (12). Nested PCR was used for specfication based on 16S rRNA gene amplification. The first round of primers used the generic gene of *Anaplasma* and *Ehrlichia*, and the second round was performed using the specific primers for the *A. phagocytophilum* (Table 1) (13). These primers were synthesized at a concentration of 10 μM (Sangon Biotech, Shanghai, China).

**Table 1:** The detection primers of *Anaplasma phagocytophilum* based on the 16S rRNA gene in nested PCR.

Primers	Primer sequence	Amplified fragments (bp)
The first round		
EH-out1	5ˊ-TTG AGA GTT TGA TCC TGG CTC AGA ACG-3ˊ	653
EH-out2	5ˊ-CAC CTC TAC ACT AGG AAT TCC GCT ATC-3ˊ	
The second round		
HGA1	5ˊ-GTC GAA CGG ATT ATT CTT TAT AGC TTG-3ˊ	389
HGA2	5ˊ-TAT AGG TAC CGT CAT TAT CTT CCC TAC-3ˊ	

The first round PCR reactions were performed in 25 μL volumes. This included 12.5 μL Dream Taq Green PCR Master Mix (K1081, Thermo Fisher Scientific, Waltham, MA, USA), 0.5 μL each of first round primers EH-out1 and EH-out2 (10 μmol/L), 5 μL DNA template, and 6.5 μL sterile deionized water. Cycling protocol was as follows: initial denaturation at 95°C for 3 min, followed by 35 cycles of denaturation at 95°C for 30 s, annealing at 55°C for 40 s, extension at 72°C for 50 s, and a final extension at 72°C for 7 min. The second round PCR reactions were performed in 30 μL volumes, including 15 μL Dream Taq Green PCR Master Mix, 0.6 μL each of second round primers HGA1 and HGA2, 0.5 μL template (the first-round PCR product), and 13.3 μL sterile deionized water. PCR conditions for the second round were the same as those for the first round, except the annealing temperature was 57°C rather than 55°C. The whole process was controlled by both positive and negative controls. Positive controls consisted of the first PCR amplified positive product for *A. phagocytophilum* confirmed by sequencing in this study, and negative controls consisted in sterile water. Second-round PCR products of 3 μL were electrophoresed on 1.5% agarose gels under the condition of 120 V for 45 min, and visualized under a Gel imaging system (G: BOX F3, Syngene, Frederick, MD, USA). The amplied products about 389 bp were confirmed as *A. phagocytophilum* positive and then sequenced in both directions by Shanghai Sangon Biotech.

### 2.3 Sequence analysis and phylogenetic analysis

The sequences were compared with published gene sequences obtained from the GenBank database, using the BLAST Sequence Similarity Searching tool.^1^ Sequences analysis was carried out using the MegAlign program in DNASTAR package. A Phylogenetic tree was constructed by a neighbor-joining method with 1,000 bootstrap replications in MEGA 7.0 software.

### 2.4 Statistical analysis

Data on geography and laboratory parameters was collected using Microsoft Excel 2016. Depending on their constituent ratio, wild small mammals were classified as dominant (>10%) or other (≤10%). In calculating the *A. phagocytophilum* infection rate, we used the number of wild small mammals infected with *A. phagocytophilum* as the numerator, and the number of wild small mammals whose DNA had been successfully extracted as a denominator. Differences of *A. phagocytophilum* infection in small mammals among species, gender, habitat, sampling seasons and counties were conducted by Fisher’s Exact Test under R software (version 4.0.1). All *p*-values were 2-tailed, and *p*<0.05 was considered statistically significant.

### 2.5 Ethics approval

The study has been approved the Medical Ethics Committee of Dali University (no. MECDU-201507-21). The small mammals captured were allowed and no painful.

## 3 Results

### 3.1 Species of wild small mammals and *A. phagocytophilum* infection

A total of 1,605 wild small mammals were collected at three counties from western Yunnan province in this study. These wild small mammals were classified into 30 species, 18 genera, six families, three orders; *Apodemus chevrieri* (29.35%, 471/1605) and *Eothenomys miletus* (19%, 305/1605) were the dominant species. In Lianghe, a total of 407 wild small mammals were identified; the dominant species were *Rattus tanezumi* (24.57%, 100/407), *Rattus steini* (19.9%, 81/407), *Mus pahari* (10.81%), and *Niviventer fulvescens* (10.07%, 41/407). Jianchuan and Yulong have the same dominant rat species. In Jianchuan, the dominant species were *Ap. chevrieri* (44.14%, 286/648), *E. miletus* (31.94%, 207/648), and *Apodemus draco* (10.65%, 69/648). In Yulong, the dominant species were *Ap. chevrieri* (33.64%, 185/550), *E. miletus* (16.55%, 91/550), and *Ap. draco* (16%, 88/550).

**Figure 2:**
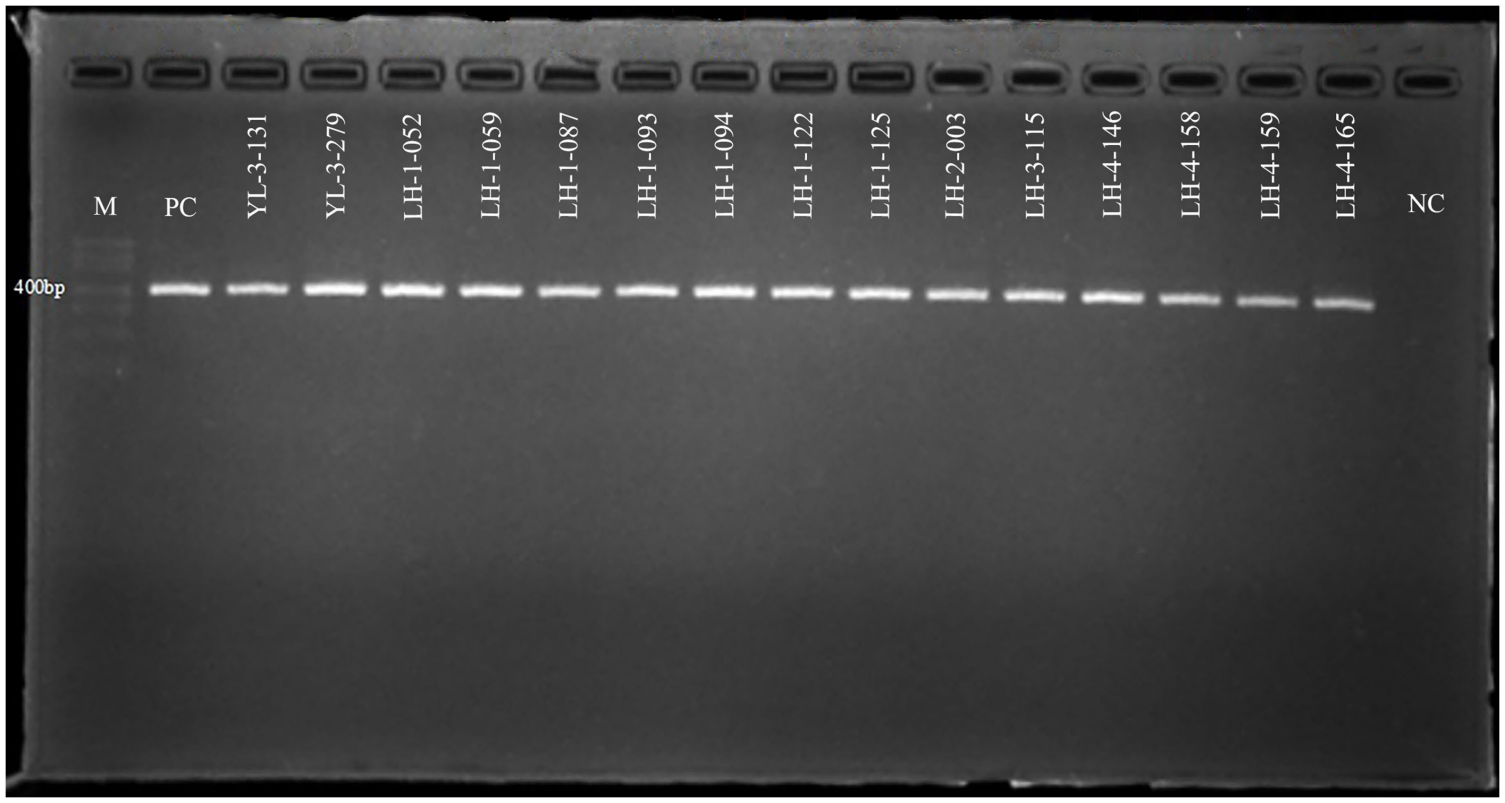
Electropherogram of *Anaplasma phagocytophilum* positive products by nested-PCR. Marker shows in the picture from top to bottom: 700, 600, 500, 400, 300, 200, 100 bp. Fifteen positive amplification products in this study were showed, which were YL-3-131 ~ LH-4-165 in turn. M, DNA marker; PC, positive control; NC, negative control; YL, Yulong; LH, Lianghe.

Fifteen out of 1,605 samples were positive according to the 16Sr RNA gene with an overall prevalence of 0.93% (Table 2). Based on the agarose gel electrophoresis results of nPCR, five of 30 species were positive with a molecular weight of about 389 bp (Figure 2). Different species showed different rates of *A. phagocytophilum* infection, ranging from 0.21 to 20%. *Rattus nitidus* showed the greastest prevalence of *A. phagocytophilum* (20.0%, 3/15), followed by *R. tanezumi* (5.77%, 6/104), *R. steini* (4.88%, 4/82), *E. miletus* (0.33%, 1/305), and *Ap. chevrieri* (0.21%, 1/471). The infection rate of *A. phagocytophilum* in wild small mammals from Lianghe and Yulong counties was 3.19% (13/407) and 0.36% (2/550), respectively. However, no positive test was found in Jianchuan (0%, 0/648). Three of the 17 species of wild small mammals were positive in Lianghe; the prevalence of *A. phagocytophilum* was highest in *R. nitidus* (21.43%, 3/14), followed by *R. tanezumi* (6%, 6/100), and was lowest in *R. steini* (4.94%, 4/81). The positive species of wild small mammals of Yulong were *E. miletus* (1.1%, 1/91) and *Ap. chevrieri* (0.54%, 1/185); the remaining species were not detected. In Jianchuan, *E. miletus* and *Ap. chevrieri* were captured in large numbers, but no positive *A. phagocytophilum* was detected (Table 2).

**Table 2:** Distribution of infection on wild small mammals captured from western Yunnan province [positive/*n* (%)].

Species	Jianchuan County	Yulong County	Lianghe County	Total
*Anourosorex aquamipes*	0/1	0	0/24	0/25
*Apodemus chevrieri*	0/286	1/185 (0.54%)	0	1/471 (0.21%)
*Apodemus draco*	0/69	0/88	0	0/157
*Apodemus latronum*	0/18	0/52	0	0/70
*Bandicota indica*	0	0	0/1	0/1
*Berylmys bowersi*	0	0	0/9	0/9
*Callosciurus erythraeus*	0	0/1	0	0/1
*Crocidura attenuate*	0/11	0/5	0/6	0/22
*Crocidura dracula*	0/3	0/6	0/3	0/12
*Dremomys pemyi*	0/1	0/20	0	0/21
*Eothenmys melanogaster*	0/2	0	0	0/2
*Eothenomys eleusis*	0	0	0/9	0/9
*Eothenomys miletus*	0/207	1/91 (1.09%)	0/7	1/305 (0.33%)
*Eothenomys proditor*	0	0/39	0	0/39
*Hylomys suillus*	0	0	0/28	0/28
*Micromys minutus*	0/6	0	0	0/6
*Mus pahari*	0	0	0/44	0/44
*Niviventer andersoni*	0/6	0/3	0/2	0/11
*Niviventer confucianus*	0/9	0/26	0/2	37
*Niviventer fulvescens*	0	0	0/41	0/41
*Rattus nitidus*	0	0/1	3/14 (21.43%)	3/15 (20%)
*Rattus norvegicus*	0/4	0	0	0/4
*Rattus steini*	0/1	0	4/81 (4.94%)	4/82 (4.88%)
*Rattus tanezumi*	0/2	0/2	6/100	6/104 (5.77%)
*Sciurotamias forresti*	0	0/1	0	0/1
*Sorex alpinus*	0	0/2	0	0/2
*Soriculus leucops*	0	0/4	0	0/4
*Suncus murinus*	0/3	0	0/32	0/35
*Tupaia belangeri*	0/19	0/23	0/4	0/46
*Vernaya fulva*	0	0/1	0	0/1
Total	0/648	2/550 (0.36)	13/407 (3.19)	15/1605 (0.93%)

### 3.2 Phylogenetic analysis based on the 16S rRNA gene of *A. phagocytophilum*

**Figure 3:**
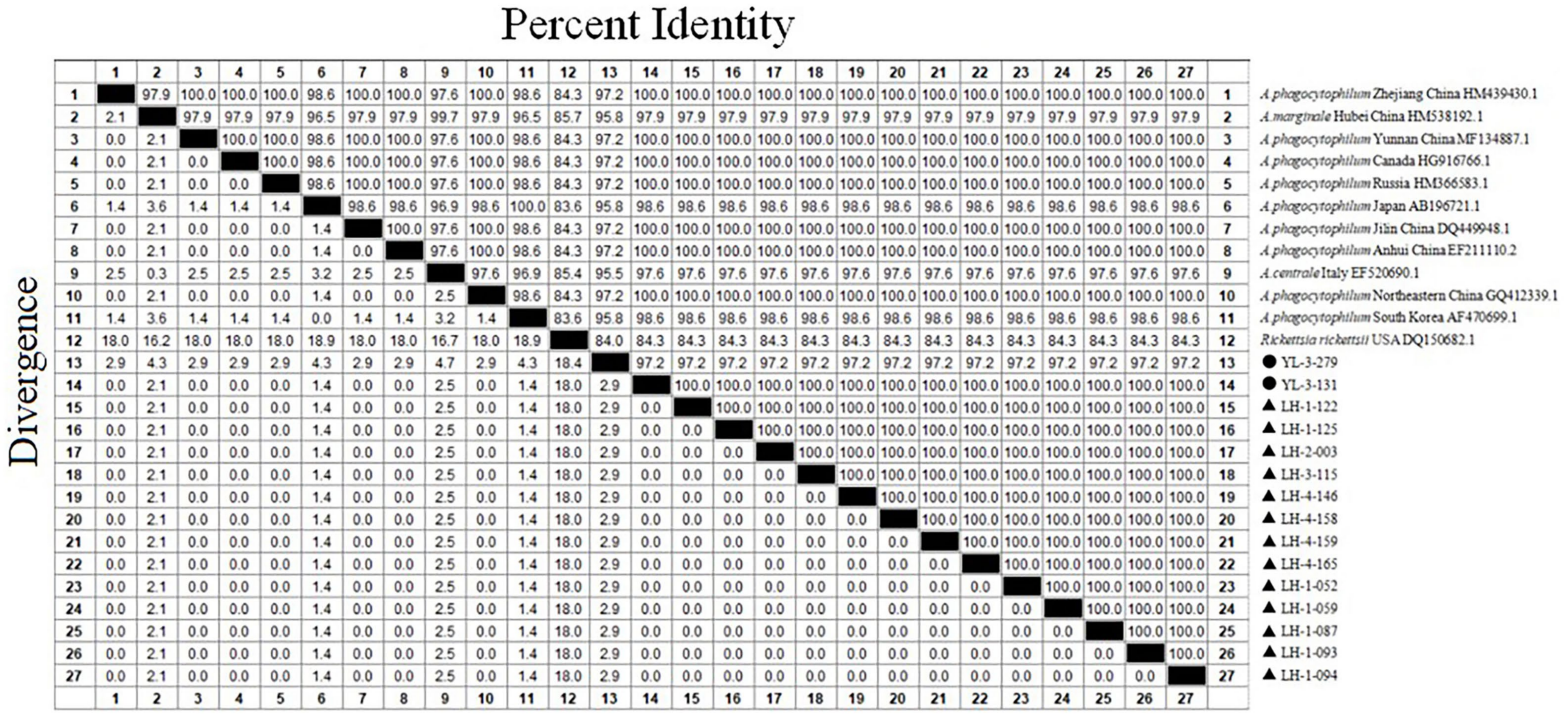
The 16S rRNA nucleotide homology matrix of *Anaplasma phagocytophilum* strain and reference strain in western Yunnan province. ▲, positive samples from Lianghe; ●, positive samples from Yulong.

Out of 15 sample sequences (13 from Lianghe, 2 from Yulong), the 16S rRNA gene nucleotide homology and phylogenetic analysis were performed. Fifteen positive sample sequences aligned with 12 reference ones, which demonstrated 84.3–100% identity to *A. phagocytophilum* strains (Figure 3). All sequences of Lianghe county (LH-1-052, LH-1-059, LH-1-087, LH-1-093, LH-1-094, LH-1-122, LH-1-125, LH-2-003, LH-3-115, LH-4-146, LH-4-158, LH-4-159, LH-4-165) were identical to each other and to a sequence from Yulong (YL-3-131). The other sequence from Yulong (YL-3-279) shared 97.2% similarity with these strains (Figure 3).

**Figure 4:**
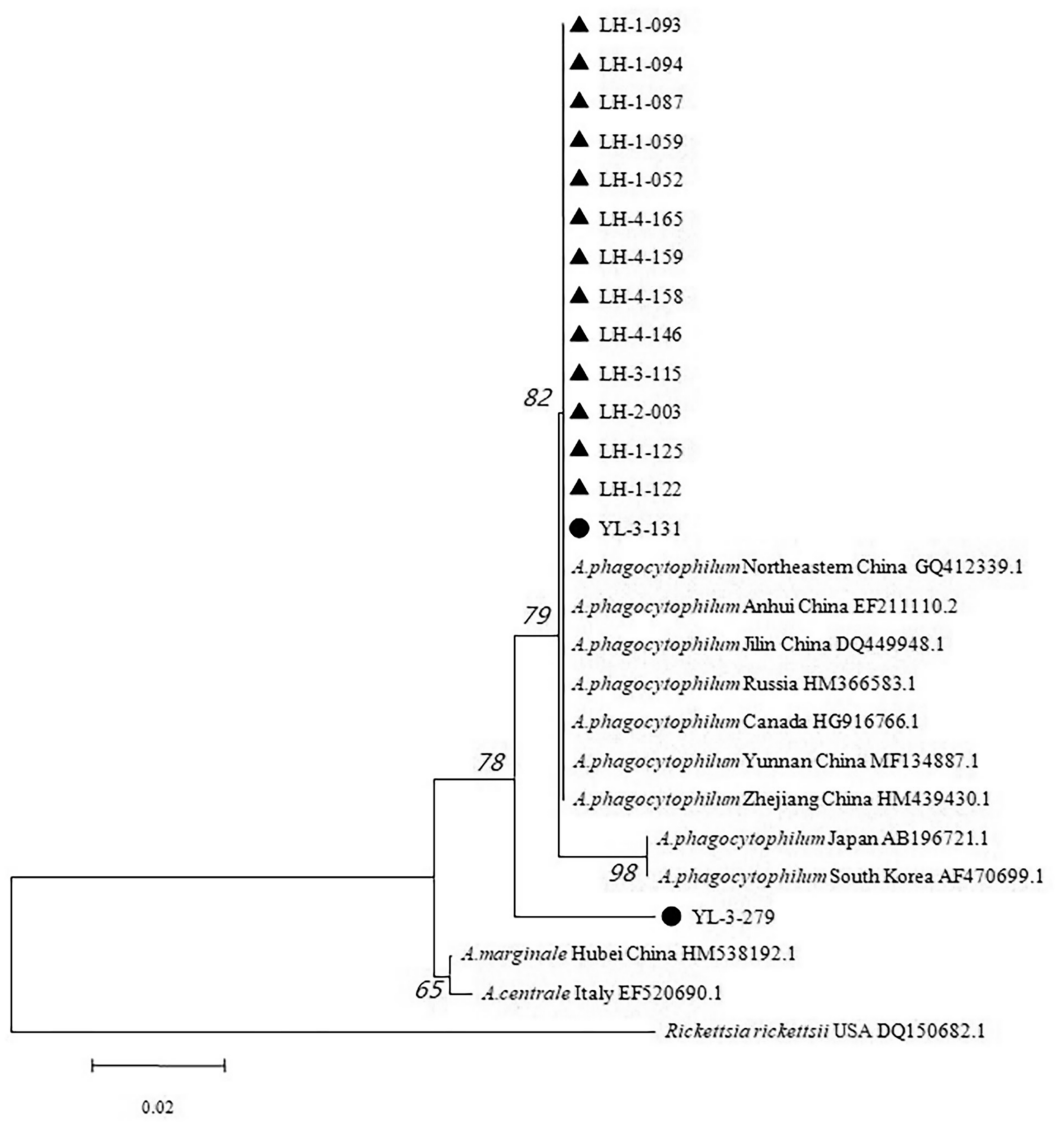
Phylogenetic tree based on 16S rRNA gene of *Anaplasma phagocytophilum*. ▲, positive samples from Lianghe; ●, positive samples from Yulong.

The phylogenetic tree revealed that all sequences from Lianghe and YL-3-131 clustered together, along with seven *A. phagocytophilum* sequences retrieved from GenBank (Anhui, Jilin, Zhejiang, Yunnan, North eastern China, Canada, and Russia). The other sequence from Yulong (YL-3-279) was clustered into an independent small branch, while *Rickettsia rickettsii* USA DQ150682.1 was at the periphery (Figure 4). The positive *R. nitidus* (3 strains), *R. tanezumi* (6 strains), *R. steini* (4 strains) all originated from Lianghe; the positive *E. miletus* (1 strains) and *Ap. chevrieri* (1 strains) all originated from Yulong. While no positive samples were found in 648 wild small mammals collected from Jianchuan.

### 3.3 Associated environmental and seasonal factors for *A. phagocytophilum* infection

The related factors of *A. phagocytophilum* infection are showed in Table 3. Compared with Yulong and Jianchuan counties, Lianghe county had a higher infection prevalence among wild small mammals (*p* < 0.001). Compared to the different species of wild small mammals, the rate of *A. phagocytophilum* infection of the dominant species is higher than the other species (*p* = 0.006). The infection rate of *A. phagocytophilum* was 1.45% in the woodland, and no infection was found in the other two habitats (Cultivated land and thickets). Nevertheless, the infection rate of *A. phagocytophilum* in wild small mammals was not significantly different in gender and sampling seasons (*p* > 0.05) (Table 3).

**Table 3:** An analysis of factors affecting *Anaplasma phagocytophilum* infection rates in wild small mammals from western Yunnan province.

Variables	Sample	Positive sample (%)	*p*-value
County			0.000
Yulong	550	2 (0.36)	
Jianchuan	648	0 (0.00)	
Lianghe	407	13 (3.19)	
Species			0.006
Dominant species^*^	776	2 (0.26)	
Other	829	13 (1.57)	
Habitat			0.010
Woodland	1,036	15 (1.45)	
Cultivated land	413	0 (0.00)	
Thickets	156	0 (0.00)	
Gender			0.297
Female	747	5 (0.67)	
Male	858	10 (1.17)	
Season			0.130
Spring	359	7 (1.95)	
Summer	368	1 (0.27)	
Autumn	469	3 (0.64)	
Winter	409	4 (0.98)	

^*^Apodemus chevrieri and *Eothenomys miletus* were the dominant species in the three counties of the western of Yunnan province in this study.

## 4 Discussion

In this study, a total of 1,605 wild small mammals were collected from three counties of the western of Yunnan province, and molecular detection and phylogenetic analysis of 15 *A. phagocytophilum* were performed. The study concerning *A. phagocytophilum* infection data from 407 wild small mammals in Lianghe County has been published in Chinese academic journals (14). Notably, we found that Lianghe County had the highest infection rate of *A. phagocytophilum*, with significant rates in *R. nitidus*, *R. tanezumi*, and *R. steini* at 20, 5.77, and 4.88%, respectively. These data highlight the potential key role of these species in the transmission of *A. phagocytophilum.*

The infection rate of *A. phagocytophilum* in Lianghe was the highest among three counties, where the 16S rRNA gene identical sequences of *A. phagocytophilum* were recovered from *R. nitidus*, *R. tanezumi*, *R. steini*, which shared 100% identity with a human case of HGA first reported in Anhui (EF211110.2) and a rodent sequence of *A. phagocytophilum* previously reported in Yunnan (MF134887.1). This result may be due to the temperature and humidity in Lianghe, which is conducive for tick growth and reproduction. Lianghe county is in the southwestern part of the Hengduan mountain, and has a rich water resource and lush vegetation due to its south subtropical monsoon climate. In the middle of the Hengduan mountain, Jianchuan and Yulong counties are separated by a low-lying valley. Yulong county has a low-latitude highland South Asian monsoon climate. Jianchuan is dominated by pine forests, which has a low-latitude highland monsoon climate. We know that geographical environment is one of the key conditions for the long-term survival of small mammals and ticks, which indirectly affects the transmission and host storage capacity of *A. phagocytophilum*. A variety of geographical conditions and climates support a wide variety of small mammals, each with its own storage capacity for *A. phagocytophilum*. It is possible that the south Asian monsoon climate in Lianghe provides ideal conditions for ticks and *A. phagocytophilum* to survive and spread. However, the association between *A. phagocytophilum* infection in wild small mammals and meteorological factors, including temperature, humidity, precipitation, and altitude, requires further investigation to elucidate the potential impacts of these environmental variables on the pathogen’s prevalence and transmission dynamics. In a word, this indicates that the potential for *A. phagocytophilum* infection in humans warrants serious consideration and underscores the necessity for enhanced surveillance of small mammals.

The genetic analysis of the two Yulong strains has elucidated a dichotomy in their phylogenetic clustering, suggesting a potential divergence in their evolutionary pathways and ecological interactions. Notably, one of the Yulong strains (YL-3-131) demonstrated a high degree of sequence similarity with the Lianghe and Anhui (EF211110.2) strains. This observation raises the possibility of zoonotic transmission to humans, considering the established propensity of the Anhui strains for human infection. The genetic proximity indicates that the Yulong strain may exhibit analogous pathogenic characteristics and transmission mechanisms, thereby necessitating a thorough investigation into its potential public health implications. In contrast, the other Yulong strain (YL-3-279) formed an independent phylogenetic branch, deviating from the genetic clustering observed for the Lianghe-like strain. The distinctive positioning of YL-3-279 in the phylogenetic tree may be attributed to two factors. One plausible explanation is the presence of a short sequence or partial mutation in the amplified product of the positive sample, which could have introduced bias in the genetic characterization. Alternatively, YL-3-279 may constitute a novel genotype of *A. phagocytophilum*, thereby illustrating the genetic diversity inherent within the species and underscoring the complexity of its evolutionary trajectory. The identification of distinct genetic variants within the Yulong strains highlights the imperative for a thorough examination of their genetic diversity. This examination should encompass the potential associations between genetic variants and their respective rodent hosts, in addition to the tick vectors implicated in their transmission. Moreover, the geographical origin of these strains may significantly influence their genetic composition, indicating a potential eco-evolutionary interaction among the pathogen, its vectors, and the local environment. Consequently, the phylogenetic analysis of the Yulong strains has revealed a complex genetic landscape that warrants further investigation. The potential for zoonotic transmission, the presence of novel genotypes, and the interactions among genetic variants, rodent hosts, tick vectors, and geographical origins necessitate a multidisciplinary approach to fully elucidate the pathogen’s biology and its public health implications.

Our present study evaluated the host, environmental factors and genetic diversity of *A. phagocytophilum* in small mammals from three counties of Yunnan in China. Previous studies demonstrated that infections with *A. phagocytophilum* occurred widely among the residents and domestic animals in Yunnan province. The seropositivity rates were 7.59% in healthy donors, 4.49% in acute undifferentiated fever patients, and 15.56% in newly enrolled college students in Yunnan province (15, 17). Another study involving nine provinces of China reported a seroprevalence of 0.6% among rural residents in Yunnan also suggested underestimating the number of reported cases (18). Another study of domestic animals showed the infection rate with *A. phagocytophilum* ranged from 6.29 to 41.08% (dogs: 6.29%; sheep: 38.10%; cattle: 41.08%) (16). Another study in Korea showed that the infection rate of *A. phagocytophilum* was 1.7% in ticks removed from humans (19). These evidences suggest that the presence of *A. phagocytophilum* in the natural environment should not be disregarded, as there exists a potential risk of transmission to humans. However, few studies on *A. phagocytophilum* infection on wild small mammals were reported in the western of Yunnan Province. The infection of *A. phagocytophilum* in small mammals in our study was 0.93% (15/1605). More importantly, the *R. nitidus*, *R. tanezumi*, and *R. steini* exhibited significantly higher infection rates of 20, 5.77, and 4.88%, respectively. These findings suggest that these wild small animals may serve as principal reservoir hosts for *A. phagocytophilum* in China. The findings also indicate the necessity of enhancing surveillance and research concerning the infection status of wild small animals in the region to remain vigilant against the potential threat they may pose to human health. Given the transmission pathways and clinical manifestations of HGA, further investigation and the implementation of preventive measures are especially critical.

Furthermore, the findings indicate that the prevalence of *A. phagocytophilum* infection in small mammals varies according to habitat type, with the highest infection rates observed in woodland areas. This variation is likely attributable to differences in tick vector density and habitat preference. Consequently, individuals engaging in activities within woodland environments should implement appropriate protective measures to minimize their risk of tick bites. No significant gender differences in the infection rates of *A. phagocytophilum* among wild small mammals were observed in our study, which contradicts the findings of previous studies. Prior studies have suggested that the infection rate of *A. phagocytophilum* is associated with both the gender and body weight of the host animals (20). The discrepancy in our results may stem from similar activity ranges and tick exposure for both male and female wild small mammals, suggesting that gender-based differences may be less significant than in previous studies due to uniform environmental interactions with the vector. The infection rate of *A. phagocytophilum* for wild small mammals did not show any significant difference between seasonal variations in this study. As is known to all, ticks are the main vectors of *A. phagocytophilum*, whose activity depends on the humidity and temperature. Seasonal changes such as rainfall or temperature have a significant impact on the host animals, the habitat and the tick vector’s feeding habits (21). A previous study imply that the ruminant and bird hosts have the characteristics of seasonal dynamics of tick-borne pathogens because of their abundance and temporal population fluctuations, but not in wild small mammals (22). This result may be explained that these tick vectors may spend all their life in their hosts’ burrows, where the environmental conditions are buffered and rely less on climatic conditions (20). Even so, seasonal changes with *A. phagocytophilum* infection should be paid more attention to avoid the occurrence and epidemic of natural focal diseases. In other words, the range of tick habitats may be expanding rapidly due to global warming, increasing the likelihood of tick-borne diseases emerging and reemerging.

This study has also some limitations. The study investigated only three counties in western Yunnan, with limited sampling points. But these three counties are rich in small mammals, which are the representative places of plague focus in Yunnan Province (Lianghe County: the commensal rodent plague focus, Jianchuan and Yulong: the wild rodent plague focus) (23). As a result of this abundance, all rodent-borne diseases may be at risk. Then, ticks are important reservoirs and vectors for *A. phagocytophilum*, and no ticks were found on the surface of the body of any small mammals during the survey. Then a comprehensive survey of the seasonal occurrence of the small mammals in consecutive years of *A. phagocytophylum* infection has not yet been carried out. It is not known about tick infestation or whether there is a correlation between tick infestation rates and small mammal infestation, which deserve further study. In addition, our study acknowledges the limitations in exploring the direct impacts of environmental variables on tick ecology and disease transmission dynamics. We recommend that future research incorporate more detailed environmental data, including long-term climate records and tick population monitoring, to better understand these complex relationships. Lastly, we did not investigate the prevalence of *A. phagocytophilum* infection in the human of this area, this also is what we are going to do next. However, several advantages exist in our study. Thirty species of small mammals were captured in this study, which were not limited to the dominant species in the area. Due to the extensive sampling of animals, this study provides a comprehensive reflection of the infection status of *A. phagocytophilum* in small mammals within the region. Secondly, the detection of *A. phagocytophilum* infection demonstrated significant disparities across spatial, species-specific, and habitat-related dimensions. These variations imply that ecological factors may play a critical role in shaping the transmission dynamics and prevalence of the pathogen. Recognizing such disparities is essential for advancing a nuanced understanding of the pathogen’s ecology and for pinpointing potential hotspots of infection. Furthermore, our research suggests the potential existence of a novel genotype of *A. phagocytophilum* within the Yulong region. This hypothesis is supported by observed genetic variations in the pathogen strains when compared to established genotypes. The identification of a new genotype could significantly enhance our understanding of the pathogen’s evolutionary history, its adaptability to diverse hosts and environments, and its potential implications for public health. Future research should utilize advanced molecular methodologies to elucidate the genetic diversity and phylogenetic relationships among various strains. These genetic insights are crucial for guiding targeted surveillance initiatives, forecasting disease transmission, and formulating effective intervention strategies.

## 5 Conclusion

This study has identified five species of wild small mammals in western Yunnan Province harboring infections of *A. phagocytophilum*, specifically *R. tanezumi*, *R. steini*, *R. nitidus*, *E. miletus*, and *Ap. chevrieri*. The genetic diversity of *A. phagocytophilum*, coupled with the observed variations in infection rates across the three sampling areas and different habitats, highlights the complex interplay between ecological factors and disease prevalence. The detection of *A. phagocytophilum* in these small mammals is significant as it indicates a potential sylvatic reservoir that could impact human health, particularly in areas where human-mammal interactions are frequent, such as woodlands. The genetic variability observed within the pathogen may influence its virulence, transmission dynamics, and the efficacy of any future intervention strategies. Given the potential for *A. phagocytophilum* to be transmitted to humans, it is imperative that public health authorities maintain vigilant surveillance of wild small mammal, particularly in ecologically diverse regions like western Yunnan Province. Continued monitoring will not only inform us about the prevalence of the pathogen but also provide insights into the effectiveness of natural barriers to pathogen transmission. In conclusion, our study provides valuable insights into the ecological factors influencing *A. phagocytophilum* infections in wild small mammals. The implications of these findings for public health are significant, warranting further research and proactive disease management strategies.

## Data Availability

The datasets presented in this study can be found in online repositories. The names of the repository/repositories and accession number(s) can be found in the article/supplementary material.

## Footnotes

^1^ http://www.ncbi.nlm.nih.gov/BLAST
